# Interleukin-2 is required for NKp30-dependent NK cell cytotoxicity by preferentially regulating NKp30 expression

**DOI:** 10.3389/fimmu.2024.1388018

**Published:** 2024-04-18

**Authors:** Nayoung Kim, Eunbi Yi, Eunbi Lee, Hyo Jin Park, Hun Sik Kim

**Affiliations:** ^1^ Department of Convergence Medicine, Asan Medical Center, University of Ulsan College of Medicine, Seoul, Republic of Korea; ^2^ Asan Institute for Life Sciences, Asan Medical Center, University of Ulsan College of Medicine, Seoul, Republic of Korea; ^3^ Department of Microbiology, Brain Korea 21 Project, Asan Medical Center, University of Ulsan College of Medicine, Seoul, Republic of Korea; ^4^ Stem Cell Immunomodulation Research Center, Asan Medical Center, University of Ulsan College of Medicine, Seoul, Republic of Korea

**Keywords:** natural killer cell, IL-2, NKp30, NKp46, acute myeloid leukemia

## Abstract

Natural killer (NK) cells are key effectors in cancer immunosurveillance, eliminating a broad spectrum of cancer cells without major histocompatibility complex (MHC) specificity and graft-versus-host diseases (GvHD) risk. The use of allogeneic NK cell therapies from healthy donors has demonstrated favorable clinical efficacies in treating diverse cancers, particularly hematologic malignancies, but it requires cytokines such as IL-2 to primarily support NK cell persistence and expansion. However, the role of IL-2 in the regulation of activating receptors and the function of NK cells expanded for clinical trials is poorly understood and needs clarification for the full engagement of NK cells in cancer immunotherapy. Here, we demonstrated that IL-2 deprivation significantly impaired the cytotoxicity of primary expanded NK cells by preferentially downregulating NKp30 but not NKp46 despite their common adaptor requirement for expression and function. Using NK92 and IL-2-producing NK92MI cells, we observed that NKp30-mediated cytotoxicity against myeloid leukemia cells such as K562 and THP-1 cells expressing B7-H6, a ligand for NKp30, was severely impaired by IL-2 deprivation. Furthermore, IL-2 deficiency-mediated NK cell dysfunction was overcome by the ectopic overexpression of an immunostimulatory NKp30 isoform such as NKp30a or NKp30b. In particular, NKp30a overexpression in NK92 cells improved the clearance of THP-1 cells *in vivo* without IL-2 supplementation. Collectively, our results highlight the distinct role of IL-2 in the regulation of NKp30 compared to that of NKp46 and suggest NKp30 upregulation, as shown here by ectopic overexpression, as a viable modality to harness NK cells in cancer immunotherapy, possibly in combination with IL-2 immunocytokines.

## Introduction

Natural killer (NK) cells are key innate immune cells with anti-tumor and anti-viral activity harnessed with multiple activating receptors, such as NKG2D, CD16 (FcγRIII), 2B4, DNAM-1, and natural cytotoxicity receptors (NCRs) (e.g., NKp30, NKp44, and NKp46) (1–4). Unlike T cells, they are not restricted by the major histocompatibility complex, and consequently do not cause graft-versus-host diseases (GvHD). In addition, NK cell alloreactivity can eliminate leukemia relapse effectively (5, 6). The safety and efficacy of NK cell therapy have been evaluated in various tumors since (7). Furthermore, recent progress in immune cell therapy with the chimeric antigen receptor (CAR) provides novel therapeutic possibilities for NK cells. Infusion of human leukocyte antigen-mismatched anti-CD19 CAR-NK cells derived from cord blood does not cause cytokine release syndrome, neurotoxicity, or GvHD in patients with CD19^+^ relapsed or refractory lymphoma or leukemia. Seventy-three percent of patients exhibited a response to treatment with these cells, and there was no increase in inflammatory cytokine levels (8). Although NK cell-based therapies have demonstrated some favorable clinical efficacies, the therapeutic benefit is largely confined to the treatment of hematologic malignancies (9). The antitumor activity of NK cells is often compromised by the immunosuppressive tumor microenvironment (TME) including tumor-derived suppressive factors, leading to tumor immune escape from immunosurveillance by NK cells (10). To enhance resistance to immunosuppression and enhance therapeutic antitumor activity, cytokines such as interleukin (IL)-2 have been employed to prime NK cells *ex vivo* and/or *in vivo* (11, 12).

IL-2 is an essential cytokine for lymphocytes, including NK cells, to survive and sustain anti-tumor function and has been approved for the treatment of metastatic melanoma and metastatic renal cell carcinoma as a form of monotherapy (11). The IL-2 receptor is made up of three subunits, IL-2Rα (CD25), IL-2Rβ (CD122), and IL-2Rγ (CD132), resulting in the formation of the high-affinity heterotrimeric receptor (IL-2Rαβγ). Previous studies on distinct NK cell subsets have revealed the expression of the high-affinity IL-2Rαβγ in CD56^bright^ NK cells and intermediate-affinity heterodimeric IL-2Rβγ in CD56^dim^ NK cells (13, 14). IL-2Rβ is shared by the IL-15 receptor, and the γ chain is common to the IL-2, 4, 7, 9, 15, and 21 receptors. IL-2Rβγ is critically involved in signaling through IL-2 and IL-15 and is required for NK cells to survive and proliferate, as a genetic deficiency of IL-2Rβ or the γ chain causes a lack of NK cells in mice (15). IL-2 induces NK cell survival through phosphatidylinositol-3 kinase activity (16). In addition to STAT1, 3, and 5, IL-2 induces STAT4 activation, resulting in an enhanced response to IL-12 for NK cell activation and proliferation (17, 18). Moreover, IL-2-activated human NK cells eliminate target cells through apoptosis and lysis (19). In support, the administration of IL-2 in patients with relapsed or refractory leukemia and metastatic solid tumors leads to an increase in NK cell numbers and cytotoxic activity (20, 21). However, IL-2 has to be administered at low or ultra-low doses for therapeutic purposes in the clinic, because of the severe toxicity including vascular leak syndrome, pulmonary edema, and cardiac toxicity, shown in the early studies with high dose administration (22–24). The *in vivo* infusion of low-dose IL-2 effectively expands NK cells whose programmed cell death is delayed in humans (25). The dose of IL-2 was 0.45 × 10^6^ IU/m^2^/d *in vivo* and 100 pM *in vitro*. In acute myeloid leukemia (AML) patients administered with low-dose IL-2 at 16,400 U/kg subcutaneously three times for 18 months, CD3^-^CD16^-^CD56^bright^ and CD3^-^CD16^+^CD56^+^ NK cells increased in the blood, and the expression of NKp30 and NKp46 also increased. Furthermore, high CD56^bright^ NK cell counts and high expression levels of NKp30 and NKp46 on CD16^+^ NK cells independently predicted leukemia-free survival and overall survival (26). Following *in vitro* culture with IL-2, the expression level of NKp30 and NKp44 was upregulated in NK cells from healthy donors (27), whereas the NKp46 expression level increased in NK cells from patients with myelodysplastic syndromes (28). These studies suggested the modulation of NK cell receptors, particularly NCRs, as a potential mechanism underlying the therapeutic efficacy of IL-2 besides its established contribution to NK cell proliferation, homeostasis, and activation.

In this study, we aimed to assess the role of IL-2 supplementation in the modulation of NK activating receptors and its effect on the antitumor activity against leukemia cells, including a xenograft mouse model of leukemia clearance. Current NK cell therapy strategies rely largely on the adoptive transfer of allogeneic or haploidentical NK cells expanded *ex vivo* and infused into the patient in the absence or presence of *in vivo* cytokine support including low-dose IL-2 (26, 29, 30). Thus, to figure out the direct impact of IL-2 on the activating receptors of clinical-grade NK cells, we used primary NK cells from healthy donors expanded *ex vivo* using cytokines (e.g., IL-2 and IL-15) and feeder cells (K562‐mb15‐41BBL), using a protocol adopted in clinical trials for hematological malignancies and solid tumors (31–33). Moreover, the findings obtained with primary expanded NK cells were validated using the NK cell line NK92. The results revealed that IL-2 deprivation, a situation that likely occurs during adoptive NK cell therapy without IL-2 support or in the TME, dampened NK cell cytotoxicity by preferentially downregulating the expression of NKp30 but not NKp46. This discordant regulation indicated a distinct effect of IL-2 on the expression of NKp30 and NKp46 despite their common requirement of FcϵRIγ and CD3ζ for surface expression and signaling (34). Furthermore, IL-2 deprivation-mediated NK cell dysfunction was overcome by NKp30 overexpression, proposing NKp30 upregulation as a viable modality to harness NK cells in cancer immunotherapy.

## Materials and methods

### Cells and reagents

Human primary samples were obtained from healthy donors after informed consent in accordance with protocols approved by the Institutional Review Board of Asan Medical Center and the Declaration of Helsinki (2018–0611). Peripheral blood mononuclear cells (PBMCs) were separated from peripheral blood using a lymphocyte separation medium (MP Biomedicals, CA, USA). Primary expanded NK cells were used as effector cells for functional assays after 24 h in the presence or absence of human recombinant IL-2 (200 U/mL; Roche, Switzerland). NK92 cells (gift of K. S. Campbell, Fox Chase Cancer Center) were cultured in a-MEM (Gibco) containing FBS (20%), MEM vitamin solution (1%; Gibco), 2-mercaptoethanol (0.1 mM; Gibco), and IL-2 (200 U/mL). NK92MI cells (American Type Culture Collection) are IL-2–independent variant of NK92 (35) and were cultured in the same medium in the absence of IL-2. Chronic myelogenous leukemia K562 (American Type Culture Collection) and mouse mastocytoma P815 (American Type Culture Collection) cells were cultured in an IMDM medium (Hyclone) containing FBS (10%). Acute monocytic leukemia THP-1 (American Type Culture Collection) cells were cultured in an RPMI medium (Gibco) containing FBS (10%) and 2-mercaptoethanol (500 μM). Plat-A retroviral packaging cells (Cell Biolabs) were cultured in DMEM (Gibco) containing FBS (10%), puromycin (1 μg/mL), and blasticidin (10 μg/mL). Recombinant human IL-15 was purchased from Peprotech (200-15), and a CSFE cell proliferation kit was obtained from Invitrogen/Thermo Fisher Scientific.

### Primary NK cell expansion

Primary human NK cells isolated from PBMCs were expanded as previously described (32), with some modifications. PBMCs (1 × 10^6^ cells) were incubated in a 24-well tissue culture plate with 100 Gy-irradiated K562-mb15-41BBL cells (1 × 10^6^ cells) in a stem cell growth medium (SCGM; CellGenix, Germany) supplemented with 10% FBS and IL-2 (10 U/mL). The half volume of the cultured medium was replaced every 2 d with a fresh medium containing IL-2 (10 U/mL). After 7 d of culture, residual T cells were depleted with a CD3 positive selection kit (StemCell Technologies). The resulting cells were incubated in an SCGM supplemented with 10% FBS, IL-2 (100 U/mL), and IL-15 (5 ng/mL; PeproTech, NJ, USA) for 2 additional weeks, with a half-medium exchange every 2 d. The CD3^-^CD56^+^ NK cells expanded 5,000-fold on average for a total of 3 weeks of culture with 96–99% purity, as assessed by flow cytometry.

### Antibodies

Antibodies for NK cell receptors and ligands were obtained from the following sources: anti-human CD337/NKp30 (210847) and anti-human CD314/NKG2D (149810) from R&D System; anti-human CD335/NKp46 (9E2) from BD Biosciences (San Jose, CA, USA); anti-human CD336/NKp44 (P44-8), anti-human CD244/2B4 (C1.7), isotype control mouse IgG1 (MOPC-21) from BioLegend; rabbit anti-human FcϵRIγ from Millipore. The following fluorochrome-conjugated Abs were used in flow cytometric analyses: anti-human CD107a-FITC (H4A3), anti-human CD3-PerCP (SK7), anti-human CD56-PE (NCAM16.2), anti-human MICA/B-PE (6D4), anti-human Nectin-2/CD112-PE (R2.525), isotype control mouse IgG1 conjugated with FITC or PE (MOPC-21), and isotype control mouse IgG2a conjugated with PE (G155-178) from BD Biosciences; isotype control mouse IgG1 conjugated with APC (MOPC-21) from BioLegend; anti-human B7-H6-PE (875001) from R&D System. The Fc receptor binding inhibitor antibody was obtained from eBioscience (San Diego, CA, USA). For the detection of signaling adaptor FcϵRIγ, NK92 cells were incubated in BD Cytofix/Cytoperm solution (BD Biosciences), blocked with 5% normal goat serum (Invitrogen), then stained using a rabbit anti-human FcϵRIγ antibody (06-727; Millipore) followed by secondary staining with goat anti-rabbit F(ab′)2-PE (Jackson ImmunoResearch, 111-116-144).

### Cytotoxic degranulation assay

The cytotoxic degranulation of NK cells was determined by measuring the cell surface expression of CD107a, as previously described (32). Briefly, effector cells were stimulated with the same number of FcR^+^ P815 cells pretreated with the antibody (10 μg/mL) raised against the indicated NK activating receptors in 96-well V-bottom culture plates (Corning Costar, NY, USA) and incubated for 2 h at 37°C. The cell pellets were resuspended in a flow cytometry buffer (PBS with 1% FBS) and stained with anti-human CD3-PerCP, anti-human CD56-PE, and anti-human CD107a-FITC antibodies for 30 min in the dark at 4°C. Lymphocytes were gated by a forward/side scatter, and the CD107a expression on CD3^-^CD56^+^ NK cells was analyzed by flow cytometry using a FACS Accuri C6 (BD Biosciences, Franklin Lakes, NJ, USA) and the FlowJo software (ver.10, Treestar, Ashland, OR, USA).

### NK cell-mediated cytotoxicity assay

The europium-based cytotoxicity assay was used to assess NK cell cytotoxicity. Briefly, target cells were loaded with a BATDA reagent (40 μM; Perkin Elmer) at 37°C for 30 min. Cells were then washed in medium containing 1 mM sulfinpyrazone (Sigma-Aldrich), resuspended at 1 × 10^6^ cells/mL in the medium, and incubated with primary expanded NK cells, NK92 cells, or NK92MI cells in the presence of sulfinpyrazone for 2 h at 37°C. The plate was then mixed and centrifuged for 5 min (1,400 rpm). The supernatant (20 μL) was then mixed with 20% europium solution (200 μL; Perkin Elmer) in acetic acid (0.3 M) for 5 min, and target cell lysis was detected on a VICTOR X4 multi-label plate reader (Perkin Elmer). For the redirected antibody-dependent cell cytotoxicity (ADCC) assay, FcR^+^ P815 cells labeled with BATDA reagent were mixed with the antibody (10 μg/mL) specific to the indicated NK activating receptors at RT for 30 min and then incubated with NK cells in the presence of sulfinpyrazone at 37°C for 2 h.

### NKp30 knockout by CRISPR/Cas9-RNP

sgRNA target sites were designed using the Cas-Design web programs (http://www.rgenome.net/). The sgRNA targets for human *NCR3* sequences are 5′-CACGTGGTTCCGAGATGAGG-3′ for sgRNA #1 and 5′-TGGGCCTTGGTGTCGGGACA-3′ for sgRNA #2. The target sequences selected had a PAM(NGG) sequence, high out-of-frame score, and no off-target sites in the genome. sgRNA molecules were synthesized with 2′-O-methyl 3′ phosphorothioate modification in the first and last three nucleotides (SYNTHEGO). To generate NKp30 knockout (KO) cells, NK92 or NK92MI cells (1.2×10^6^ cells) were resuspended in an Amaxa solution R (Lonza, VCA-1001; 100 μL) with a mixture of RNP including 500 pmol of sgRNA, Cas9 (IDT, 1081061) and enhancer (IDT, 1075915), and then transfected under the program A-024 using the Amaxa Nucleofector II system. Cells were then seeded, incubated in the culture media with IL-2 (200 U/mL for NK92) for 48 h, and then assayed as indicated.

### Generation of NK92 and NK92MI cells overexpressing NKp30

To produce a retrovirus for NKp30 overexpression, cDNA sequences for two NKp30 immunostimulatory isoforms (e.g., NKp30a and NKp30b) were synthesized using GenScript (Piscataway, NJ, USA). The coding sequences for NKp30a and NKp30b were cloned into the pMX-IRES-EGFP retroviral vector (System Biosciences, USA). For transduction, Plat A cells were transfected with the retroviral vector (pMX-IRES-EGFP, pMX-IRES-NKp30a-EGFP, or pMX-IRES-NKp30b-EGFP) using X-tremeGENE 9 (Roche, 50730400). After 24 h of media change, the supernatant containing virus was harvested for 24 h, mixed with a fresh medium at a 1:1 ratio, and polybrene (10 *μ*g/mL) and IL-2 (200 U/mL) were added to make a transducing mix. NK92 or NK92MI cells (1 × 10^6^) with an NKp30 KO (ΔNCR3) were resuspended in the transducing mix (2.4 mL) and then transferred to a 12-well plate. The plate was centrifuged at 700 ×g for 30 min at 32 °C and incubated for 3 h. After another centrifugation at 700 ×g for 30 min at 32 °C and 6 h of incubation, cells were washed and resuspended with fresh medium. Three days post-transduction, cells with a matched level of GFP expression were selected using a FACS Aria cell sorter. NKp30 expression was confirmed by cell surface staining using an anti-NKp30-PE (clone Z25; Beckman Coulter) antibody and reverse transcription-polymerase chain reaction (RT-PCR) to confirm isoform expression.

### RT-PCR

Total RNA was isolated from wild-type or transduced NK92 and NK92MI cells using the Trizol RNA isolation reagent (Invitrogen) according to the manufacturer’s instructions to determine the isoform-specific mRNA expression of NKp30. Briefly, 1 μg of RNA was synthesized into cDNA using a ReverTraAce™ qPCR-RT kit (TOYOBO, Japan). PCR was performed by repeating 30 cycles of 5 min at 95°C, 30 s at 94°C, 30 s at 60°C, 1 min at 72°C, and 10 min at 72°C using the following primers: Universal NKp30-F: 5′-GCAACGGCACACGGCTGGTG-3′ (forward), NKp30A: 5′-TCAGCCGCCGGGCACAGG-3′ (reverse for NKp30a), NKp30B: 5′-TCACAGCTGGGGGAAGCCGG-3′ (reverse for NKp30b), and NKp30C: 5′-TCAGGGACATCTAGGCTCTGGGATC-3′ (reverse for NKp30c). The gene expression level was normalized to β-actin expression in the control group.

### *In vivo* leukemia clearance assay

To assess whether NKp30 overexpression affects cancer surveillance by NK cells *in vivo*, we used a lymphoma clearance assay (36, 37), modified to assess the killing of THP-1 AML cells by NK cells in the immune-deficient mice. Briefly, 8-week-old NOD.Cg-Prkdc^scid^ Il2rg^tm1Wjl^/SzJ (NSG) mice lacking T, B, and NK cells were purchased from JA BIO (Suwonsi, Gyeonggido, Korea) and injected intraperitoneally (i.p.) with THP-1 target cells (5 × 10^6^ cells) labeled with CFSE (3 μM). Thereafter, the vehicle, NK92-WT, or ΔNCR3+NKp30a cells were i.p. injected into NSG mice 15 min post-injection of THP-1 cells (10 × 10^6^ cells) at an E:T ratio of 2:1. After 24 h, peritoneal cells were collected and analyzed by flow cytometry for the clearance of CFSE-stained THP-1 cells. In addition, recovered cells were stained with CD56-APC for the selective labeling of NK92 cells to facilitate the identification of THP-1 cells (CFSE^+^CD56^-^) relative to NK92 cells (CFSE^-^CD56^+^). All experimental protocols were approved by the Institutional Animal Care and Use Committee of the Asan Institute for Life Sciences.

### Statistical analysis

Each experiment was performed in duplicate or triplicate and repeated independently at least three times. Two groups were compared using two-tailed Student’s t-tests or Mann-Whitney U test. Differences between multiple groups were analyzed using one-way analysis of variance (ANOVA) or two-way ANOVA. All data were analyzed using GraphPad Prism software (ver.7.00, GraphPad Software, Inc., San Diego, CA, USA). Statistical significance was defined as p < 0.05, and the degree of significance is indicated as follows: *P < 0.05, **P < 0.01, ***P < 0.005, and **** *P* < 0.001.

## Results

### IL-2 deprivation downregulates NKp30 expression in primary expanded NK cells

**Figure 1:**
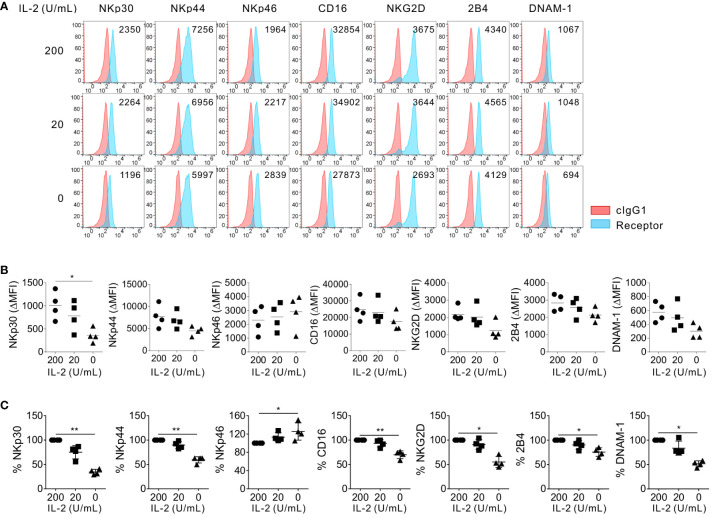
Preferential effect of IL-2 on the surface expression of NKp30. Primary expanded NK cells were incubated with the indicated dose of IL-2 (0, 20, or 200 U/mL) for 24 h, and the surface expression of NKp30, NKp44, NKp46, CD16, NKG2D, 2B4, and DNAM-1 on NK cells was determined by flow cytometry (*n* = 4 per group). **(A)** Representative FACS profiles showing the mean fluorescence intensity (MFI) of the indicated receptor expression (blue shaded histograms) on NK cells. Isotype control staining is shown as red shaded histograms. **(B)** Summary graphs showing the MFI of the indicated receptors on NK cells relative to the MFI of the isotype control (ΔMFI). Horizontal bars denote the medians. **(C)** Shown are the normalized levels for the expression of the indicated receptors. [% MFI = (ΔMFI of each condition/ΔMFI of IL-2 200 U/mL) × 100]. Horizontal bars denote the medians. *P < 0.05 and **P < 0.01; One-way ANOVA test. Each data point represents a single donor.

To address the role of IL-2 in the modulation of NK activating receptors, we assessed the effect of IL-2 supplementation or deprivation on the surface expression of diverse NK activating receptors, such as NKp30, NKp44, NKp46, CD16, NKG2D, 2B4, and DNAM-1. Primary NK cells from healthy donors were expanded according to the protocol adopted in clinical trials and used as effector cells (31, 32). The primary expanded NK cells were further incubated in the absence or presence of different IL-2 concentrations (200 U/mL or 20 U/mL) for 24 h (**Figure 1**). The flow cytometric analysis of the surface NK activating receptors revealed that the NK activating receptors tested were not significantly modulated but rather maintained by IL-2 supplementation at both high dose (200 U/mL) and low dose (20 U/mL). In contrast, NKp30 expression was significantly downregulated in mean fluorescence intensity (MFI) and percentages (%) by IL-2 deprivation, compared to that of NK cells supplemented with IL-2 (**Figures 1B, C**). The MFI and the percentage of NKp30^+^ NK cells were reduced by <50% (**Figure 1B**). In comparison, the expression levels (MFI) of NKp44, CD16, NKG2D, 2B4, and DNAM-1 on NK cells were moderately but not significantly downregulated by IL-2 deprivation. Moreover, the MFI of NKp46 on NK cells was rather upregulated by IL-2 deprivation. The percent population of NKp44^+^, CD16^+^, NKG2D^+^, 2B4^+^, and DNAM-1^+^ NK cells significantly decreased but to a lesser extent than that of NKp30^+^ NK cells by IL-2 deprivation, whereas the percentage of NKp46 expression did not decrease but rather increased on the same NK cells (**Figure 1C**). Thus, these results indicated the downregulation of NK activating receptors except NKp46 by IL-2 deprivation with the most significant effect on NKp30. IL-2 and IL-15 receptors share the β subunit and common γ chain and play pivotal roles in T and NK cell activation and homeostasis (38). In support, we observed that IL-15 deprivation for 24 h significantly reduced the MFI of 2B4, DNAM-1, and most notably NKp30, as well as the positive percentages of diverse activating receptors, particularly NKp30 on primary expanded NK cells from healthy volunteers (**Supplementary Figure S1**). These results indicated similar effects of IL-2 and IL-15 deprivation on the downregulation of activating receptors, particularly NKp30.

### NKp30-dependent NK cytotoxicity is impaired by IL-2 deprivation

**Figure 2:**
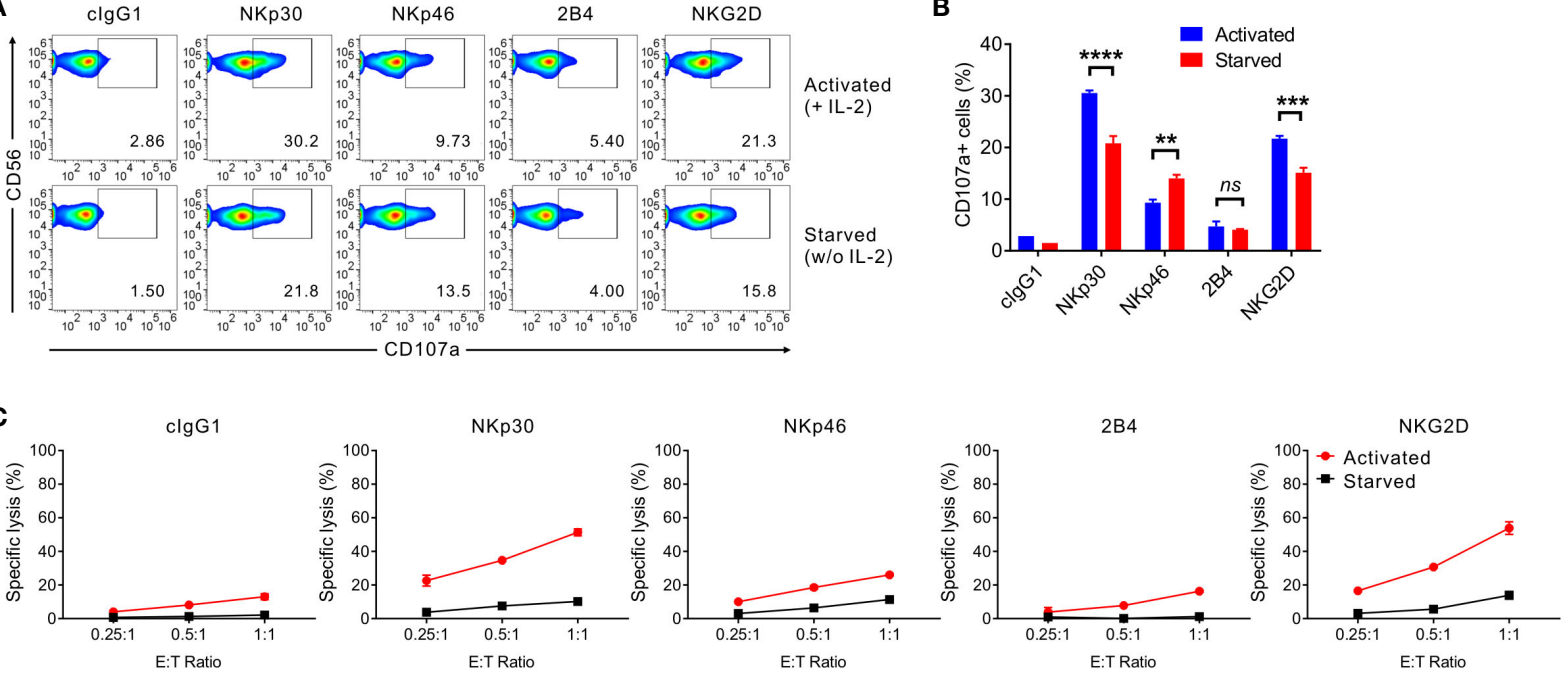
IL-2 depletion leads to reduced NKp30-dependent NK cell cytotoxicity. **(A, B)** Primary expanded NK cells were incubated in the absence (starved) or presence of IL-2 (200 U/mL; activated) for 24 h and mixed with P815 cells preincubated with isotype control antibody (cIgG1) or antibodies to the indicated receptors in the presence of the fluorochrome-conjugated anti-CD107a antibody. After incubation for 2 h, the cells were stained with the fluorochrome-conjugated anti-CD56 antibody, and the level of CD56+CD107a+ NK cells was measured using flow cytometry. Representative FACS profiles **(A)** and summary graph **(B)** showing the percentage of CD107a+ NK cells. Data are shown as the mean ± SD. **P < 0.01, ***P < 0.005, **** *P* < 0.001; Student′s *t*-test. **(C)** Primary expanded NK cells were incubated with (activated) or without (starved) IL-2 (200 U/mL) for 48 h and mixed with P815 cells preincubated with the cIgG1 antibody or antibodies to the indicated receptors. After incubation for 2h, NK cell cytotoxicity was assessed using a europium-based cytotoxicity assay at the indicated effector to target (E:T) cell ratios. Data are shown as the mean ± SD. Data are representative of at least four independent experiments with different donors. ns means, not significant.

The effect of IL-2 deprivation on the cytotoxic activity of primary expanded NK cells was assessed in redirected ADCC by CD107a degranulation and a europium assay. To this end, we used FcR^+^ P815 target cells that bind antibodies via the Fc-region and, upon incubation with antibodies to NK receptors, activate NK cells through specific activating receptors in direct cell-cell contact (32). We assessed the effect of IL-2 deprivation on cytotoxic activity through NK activating receptors, such as NKp30 and NKG2D (significant downregulation), NKp46 (marginal upregulation), and 2B4 (marginal downregulation). As seen in **Figure 2**, the NKp30- and NKG2D-mediated cytotoxic activity were significantly reduced by IL-2 deprivation for 24 h, correlating with the cognate receptor downregulation. The dependence of NKG2D-mediated cytotoxicity on IL-2 deprivation was compatible with a previous study of the coupling of NKG2D signaling to IL-15 receptor signaling (39). The expression level of CD107a, a widely used marker for cytotoxic degranulation, on NK cells decreased by over 30% in NKp30-mediated stimulation, while it decreased by approximately 25% in NKG2D-mediated stimulation (**Figures 2A, B**). Nonetheless, NKp46-mediated degranulation was upregulated, and 2B4-mediated degranulation was not significantly affected by IL-2 deprivation. The results were validated by the specific lysis of P815 target cells engaging specific activating receptors with a noticeable effect on the cytotoxicity mediated by NKp30 and NKG2D stimulation (**Figure 2C**). These results suggested that IL-2 deprivation significantly affects the cytotoxic degranulation of NK cells and the lysis of target cells through stimulation of NKG2D and, particularly, NKp30.

**Figure 3:**
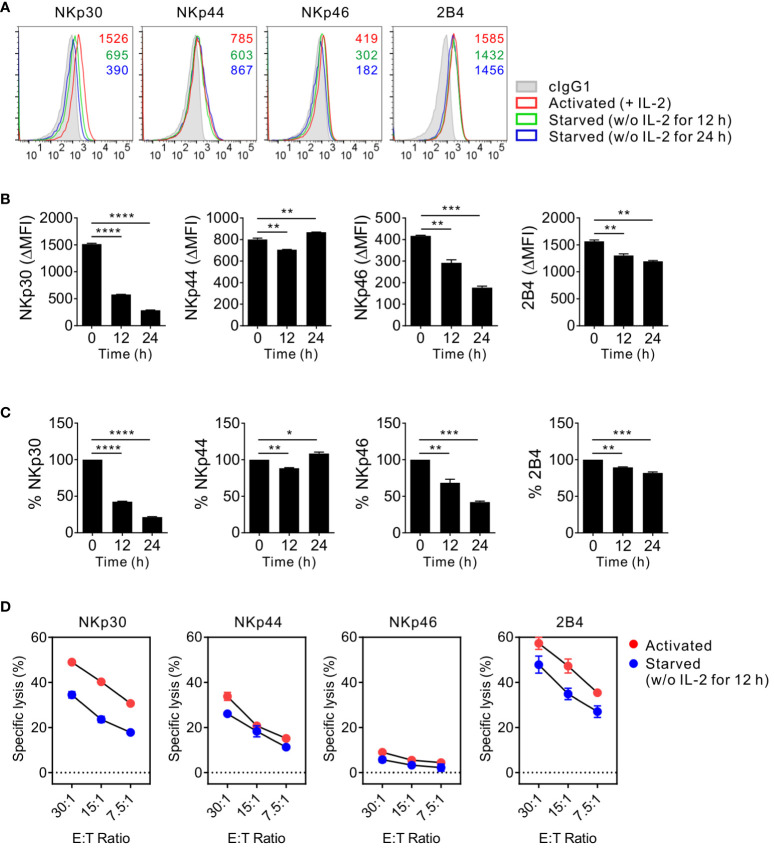
Conserved effect of IL-2 on NKp30 expression and NKp30-dependent cytotoxicity in NK92 cells. **(A)** NK92 cells were incubated with (activated) or without (starved) IL-2 (200 U/mL) for 12 h or 24 h and were used to determine the surface expression of NKp30, NKp44, NKp46, and 2B4 by flow cytometry. Representative FACS profiles showing the MFI of the indicated receptor expression (open histograms) on NK92 cells relative to the MFI of the isotype control (ΔMFI). Isotype control staining is shown as gray shaded histograms. **(B)** Summary graphs showing the MFI of the indicated receptors on NK cells relative to the MFI of the isotype control (ΔMFI). Data are shown as the mean ± SD. **(C)** Shown are the normalized levels for the expression of the indicated receptors. [% MFI = (ΔMFI of each condition/ΔMFI of IL-2 200 U/mL) × 100]. Data are shown as the mean ± SD. **(D)** NK92 cells were incubated with (activated) or without (starved) IL-2 (200 U/mL) for 12 h and mixed with P815 cells coated with antibodies to the indicated receptors. NK cell cytotoxicity was determined after 2 h using europium-based assay at the indicated effector to target (E:T) cell ratios. Data are shown as the mean ± SD. *P < 0.05, **P < 0.01, ***P < 0.005, ****P < 0.001; One-way ANOVA test. Data are representative of at least three independent experiments.

Next, to avoid donor variation in receptor profiles and responses of primary NK cells and validate the results obtained with primary NK cells, we used NK92 cells, an immortalized human NK cell line currently used in clinical trials (29, 31) (**Figure 3**). Similar to primary NK cells, NK92 cells require IL-2 supplementation for maintenance and expansion. The surface expression of NKp30, NKp44, and 2B4 but not NKp46 decreased due to IL-2 deprivation for 12 h and decreased further for 24 h starvation (**Figures 3A-C**). In particular, the expression level of NKp30 significantly decreased by > 55%, whereas that of other receptors decreased by 10-32% following IL-2 deprivation for 12 h. The consistent and preferential downregulation of NKp30 but moderate downregulation of NKp46 in NK92 cells suggested a subtle difference between primary NK cells and NK92 cells. We next assessed the cytotoxic activity of NK92 cells via NK activating receptors upon IL-2 deprivation for 12 h due to a substantial increase in apoptosis for 24 h starvation (data not shown). The specific lysis of P815 target cells mediated by NKp30 was also most notably downregulated by IL-2 deprivation (**Figure 3D**). The results suggested that NKp30 expression and corresponding NK cytotoxicity rely largely on IL-2 supplementation and that the mechanism of IL-2-dependent NKp30 regulation is conserved in the primary NK and NK92 cells. Further, we assessed the expression levels of FcϵRIγ upon IL-2 deprivation, given its requirement for surface expression of NKp30 and NKp46 (34). FcϵRIγ expression was moderately decreased in MFI by IL-2 deprivation (**Supplementary Figure S2**), compatible with a recent study of IL-2-mediated upregulation of FcϵRIγ (40). In this situation, we observed a gradual decrease in the expression level of NKp46 but a considerable decrease in NKp30 expression level, suggesting a distinct mechanism of regulation between NKp30 and NKp46 by IL-2 deprivation.

### NKp30 deletion diminishes the corresponding cytotoxicity of NK92 cells

**Figure 4:**
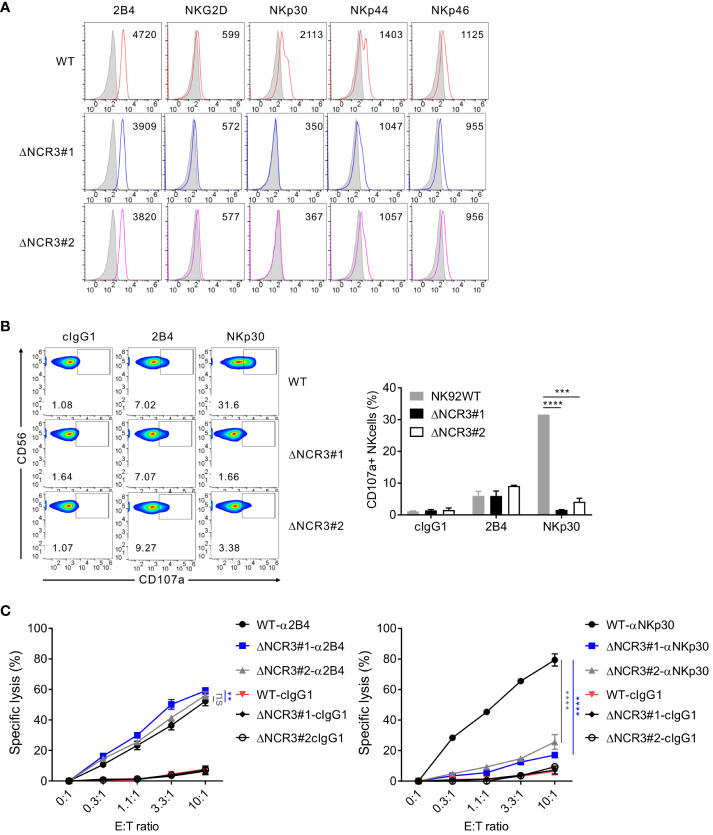
Generation of CRISPR/Cas9-mediated knockout of NKp30 in NK92 cells. **(A)** Validation of the NKp30 knockout in NK92 cells. NK92 control wild-type (WT; CRISPR/Cas9-mediated control group) and NK92 NKp30 KO cells (ΔNCR3#1 or ΔNCR3#2; CRISPR/Cas9-mediated NKp30 knockout group) were harvested, and the surface expression of 2B4, NKG2D, NKp30, NKp44, and NKp46 was determined by flow cytometry. Shown is the representative FACS profiles with the MFI of the indicated receptor expression (open histograms). Isotype control staining is shown as gray shaded histograms. **(B)** NK92 WT or NKp30 KO cells were incubated with P815 cells preincubated with cIgG1 antibody or antibodies to 2B4 or NKp30 in the presence of a fluorochrome-conjugated anti-CD107a antibody for 2 h Thereafter, cells were stained with a fluorochrome-conjugated anti-CD56 antibody, and the level of CD56+CD107a+ NK cells was measured using flow cytometry. Representative FACS profiles (*left*) and a summary graph (*right*) showing the percentage of CD107a+ NK cells. Data are shown as the mean ± SD. **(C)** Lysis of P815 cells by NK92 WT or NKp30 KO cells at the indicated effector to target (E:T) cell ratios. Cytotoxicity against P815 cells preincubated with the antibody specific for 2B4 (*left*) or NKp30 (*right*) was determined after 2 h with a europium-based assay. Data are shown as the mean ± SD. ns > 0.05, **P < 0.01, ***P < 0.005, ****P < 0.001; Student′s *t*-test **(B)** and one-way ANOVA test **(C)**. Data are representative of at least three independent experiments.

To identify the importance of NKp30 in target cell lysis by NK cells, the CRISPR/Cas9 system was used to produce a series of deletion mutants of *NCR3*, the NKp30 gene, in NK92 cells. The two common guide RNA sequences in exon 2 of *NCR3* were targeted to delete all six NKp30 variants (**Supplementary Figure S3**). The corresponding two NKp30-KO NK92 cell clones were selected and named ΔNCR3#1, where the #1 sequence was targeted, and ΔNCR3#2, where the #2 sequence was targeted. Phenotypic analysis of the KO NK92 cells (ΔNCR3#1 and ΔNCR3#2) revealed that the NKp30 expression level robustly decreased but that the expression of other activating receptors such as 2B4, NKG2D, NKp44, and NKp46 was not significantly affected as expected (**Figure 4A**). Accordingly, the KO cells (ΔNCR3#1 and ΔNCR3#2) failed to degranulate in response to NKp30 but degranulated normally in response to 2B4 (**Figure 4B**). The results were confirmed by the selective abrogation of the specific lysis of P815 target cells mediated by NKp30 but not 2B4 (**Figure 4C**). To assess the dependence of NKp30-mediated cytotoxic function on IL-2, we also produced the NKp30 KO cells in NK92MI cells, an IL-2-independent human NK cell line engineered to produce IL-2 (35). We confirmed that the IL-2-independent NK92MI cells exhibit growth rate and cytotoxicity comparable to or better than NK92 cells cultured in the medium supplemented with IL-2 (data not shown). The selective abrogation of NKp30-mediated cytotoxic activity was reproduced in NK92MI cells with NKp30 KO (data not shown), indicating the suitability of these KO cells in the assessment of NKp30-mediated cytotoxic activity irrespective of IL-2 support. Overall, the results suggest the fundamental role of NKp30 in mediating cytotoxicity by NK92 cells.

### Forced NKp30 expression rescues the reduced cytotoxicity by IL-2 deprivation

We then evaluated NK92 cell cytotoxicity against K562 chronic myeloid leukemia cells and THP-1 acute monocytic leukemia cells, given their expression of B7-H6, a ligand for NKp30 (41, 42), and NKp30 as a prognostic biomarker in AML (26, 43, 44). The constitutive expression of B7-H6 on both target cells was observed with high expression on K562 cells and moderate expression on THP-1 cells (**Supplementary Figure S4**). In the absence of IL-2 supplementation, the cytotoxicity of wild-type NK92 cells against K562 and THP-1 cells was remarkably reduced in a time-dependent manner; the effect of IL-2 deprivation was most pronounced at 24 h and also potent at 12 h (**Supplementary Figure S5A**). Likewise, a similar decrease in the cytotoxicity of primary expanded NK cells against K562 and THP-1 cells was observed following IL-2 deprivation (**Supplementary Figure S5B**). These results suggested that IL-2 deprivation significantly affects the cytotoxicity of NK cells against NKp30 ligand-expressing target cells, in accordance with the dependence of NKp30 expression and function on IL-2.

**Figure 5:**
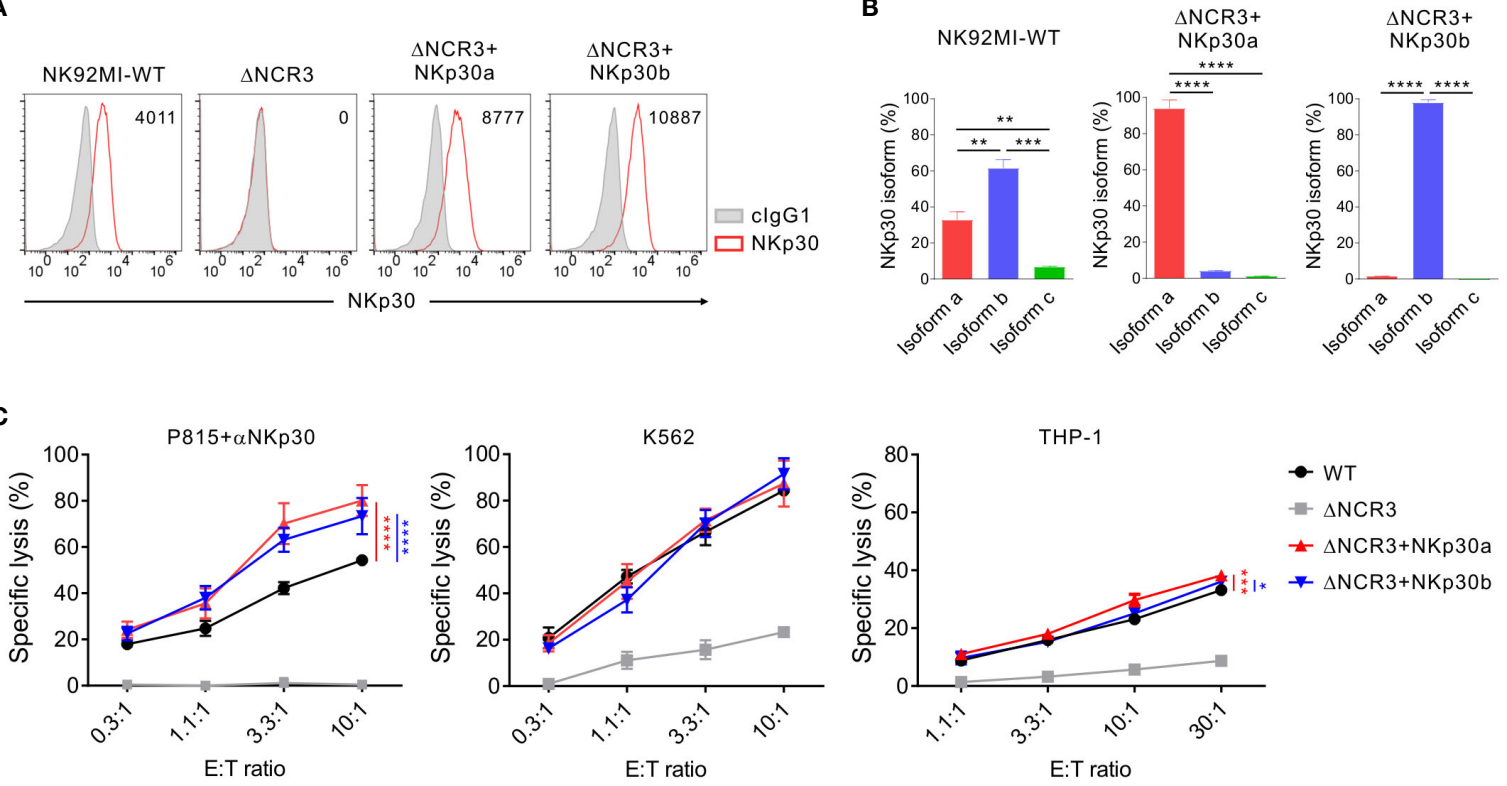
Marginal effect of NKp30 overexpression on NKp30-dependent cytotoxicity by IL-2-expressing NK92MI cells. **(A)** Validation of NKp30 overexpression in NK92MI cells. NK92MI control WT, NKp30 KO cells (ΔNCR3), and NKp30 KO cells expressing isoform a (ΔNCR3+NKp30a) or isoform b (ΔNCR3+NKp30b) were harvested, and the surface expression of NKp30 was determined by flow cytometry. Shown are the representative FACS profiles with the MFI of NKp30 expression (open histograms) relative to the MFI of the isotype control (ΔMFI). Isotype control staining is shown as gray shaded histograms. **(B)** Relative mRNA levels of NKp30 isoforms from NK92MI WT, ΔNCR3+NKp30a, or ΔNCR3+NKp30b cells were determined by using qRT-PCR and normalized to GAPDH mRNA. **(C)** Lysis of P815, K562, or THP-1 cells by NK92MI WT, ΔNCR3, ΔNCR3+NKp30a, and ΔNCR3+NKp30b cells at the indicated effector to target (E:T) cell ratios. Cytotoxicity against P815 cells preincubated with anti-NKp30 antibody (*left*), K562 (*middle*), or THP-1 (*right*) was determined after 2 h with a europium-based assay. Data are shown as the mean ± SD. *P < 0.05, **P < 0.01, ***P < 0.005, ****P < 0.001; One-way ANOVA test **(B)** and two-way ANOVA test **(C)**. Data are representative of at least three independent experiments.

The next experiments were designed to investigate whether the debilitated NK cell cytotoxicity by IL-2 deprivation could be rehabilitated by ectopic NKp30 overexpression. To this end, NK92MI and NK92 cells with the NKp30 KO (ΔNCR3) were transduced with retroviruses that express NKp30a or NKp30b, two immunostimulatory isoforms of NKp30, rather than NKp30c, an immunosuppressive isoform (34, 45). We first assessed the effect of forced NKp30 expression on the cytotoxicity of IL-2-independent NK92MI cells (**Figure 5**). We confirmed the cell surface expression of NKp30 in transduced NK92MI cells (ΔNCR3+NKp30a or ΔNCR3+NKp30b) compared with NK92MI cells with NKp30 KO (ΔNCR3) (**Figure 5A**). By employing NKp30 isoform-specific qPCR, we validated that the forced expression of NKp30a or NKp30b did not affect the expression of other isoforms (**Figure 5B**). In particular, the expression of NKp30c, the immunosuppressive isoform, was also not affected. The overexpression of NKp30a or NKp30b improved the cytotoxicity of NK92MI cells in redirected ADCC targeting NKp30 compared with wild-type NK92MI cells (**Figure 5C**). However, the cytotoxicity of NK92MI cells against K562 and THP-1 cells was marginally improved by the same forced expression of NKp30 (**Figure 5C**). These results suggested that sufficient IL-2 supplementation, as observed in IL-2 producing NK92MI cells, could enhance the function of other NK activating receptors and likely negate the effect of NKp30 overexpression.

**Figure 6:**
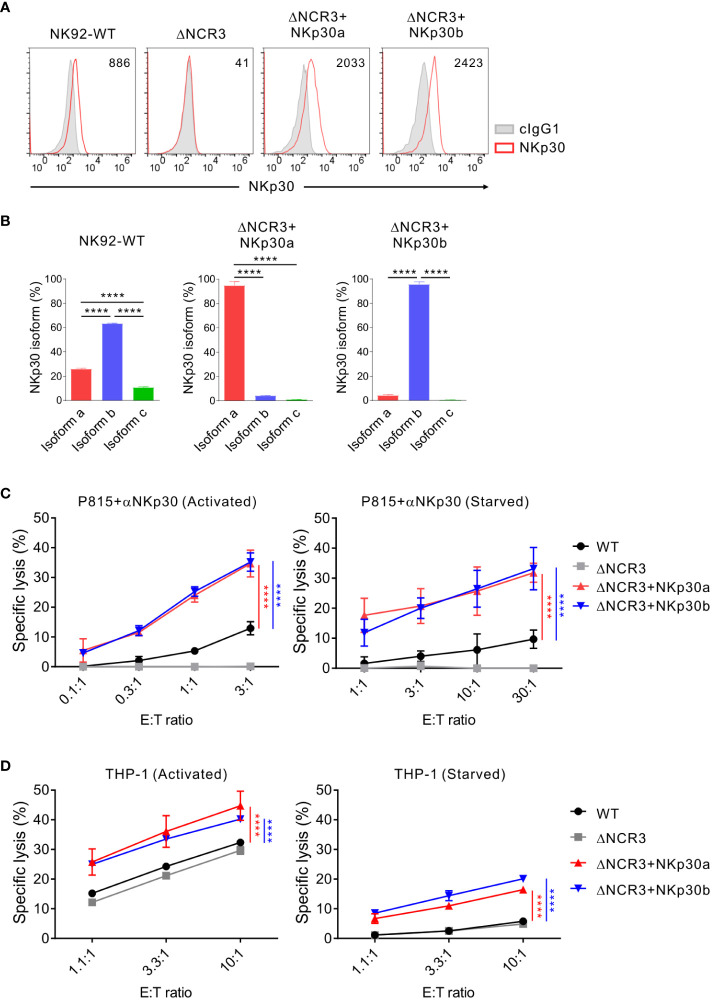
NKp30 overexpression overcomes the IL-2 depletion-mediated dysfunction of NK cells. **(A)** Analysis of NKp30 surface expression on NK92-WT, NKp30 KO cells (ΔNCR3), and NKp30 KO cells expressing NKp30a (ΔNCR3+NKp30a) or NKp30b (ΔNCR3+NKp30b) by flow cytometry. Shown are the representative FACS profiles with the MFI of NKp30 expression (open histograms) relative to the MFI of the isotype control (ΔMFI). Isotype control staining is shown as red shaded histograms. **(B)** Relative mRNA levels of NKp30 isoforms from NK92-WT, ΔNCR3+NKp30a, or ΔNCR3+NKp30b cells were determined by using qRT-PCR and normalized to GAPDH mRNA. **(C, D)** NK92-WT, ΔNCR3, ΔNCR3+NKp30a, and ΔNCR3+NKp30b cells were incubated with (activated) or without (starved) IL-2 (200 U/mL) for 24 h and mixed with P815 cells preincubated with the anti-NKp30 antibody **(B)** or THP1 cells **(C)**. NK cell cytotoxicity was determined after 2 h using a europium-based assay at the indicated effector-to-target (E:T) cell ratios. Data are shown as the mean ± SD. ****P < 0.001; One-way ANOVA test **(B)** and two-way ANOVA test **(C, D)**. Data are representative of at least three independent experiments.

We then performed the same experiments with NK92 cells that do not produce IL-2 to validate the importance of IL-2 deprivation in mediating NK cell cytotoxicity against K562 and THP-1 cells. The cell surface expression of NKp30 was confirmed in transduced NK92 cells (ΔNCR3+NKp30a or ΔNCR3+NKp30b) compared with NKp30-KO NK92 cells (ΔNCR3) (**Figure 6A**). We also validated no effect of the NKp30a or NKp30b overexpression on the expression of other isoforms including NKp30c using NKp30 isoform-specific qPCR (**Figure 6B**). The forced expression of NKp30a or NKp30b noticeably increased the cytotoxicity of NK92 cells in the presence of IL-2 in redirected ADCC targeting NKp30 and in specific lysis against THP-1 (**Figures 6C, D**, left panels). Even in the absence of IL-2 for 24 h, the forced expression of NKp30a or NKp30b not only rescued the impaired cytotoxicity in redirected ADCC targeting NKp30 but also rescued the specific lysis against THP-1 cells (**Figures 6C, D**, right panels). Moreover, NKp30 overexpression improved NK92 cell cytotoxicity against K562 target cells in the presence or absence of IL-2 supplementation; the effect of the forced expression of NKp30a or NKp30b on K562 cytolysis was most notable by IL-2 deprivation (**Supplementary Figure S6**). Collectively, these results imply that IL-2 deprivation may dampen the outcomes of NK cell therapy for leukemia patients with a significant effect on NKp30 expression and function, which could be circumvented by the overexpression of a stimulatory NKp30 isoform.

### NKp30 overexpression enhances NK cell-mediated antitumor effect *in vivo*

**Figure 7:**
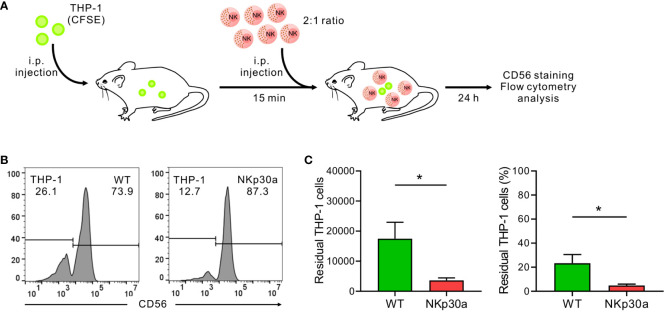
NKp30 overexpression enhances NK cell-mediated protection against THP-1 cells *in vivo*. **(A)** *In vivo* leukemia rejection protocol. Immunodeficient NSG mice lacking T, B, and NK cells received an intraperitoneal (i.p.) injection with THP-1 target cells labeled with CFSE. Thereafter, the vehicle, NK92-WT cells, or ΔNCR3+NKp30a cells were i.p. injected into NSG mice (*n* = 5 per group) 15 min post-injection of THP-1 cells at an E:T ratio of 2:1. The rejection of THP-1 cells in the peritoneal cavity was measured by flow cytometry after 24 h **(B, C)** Flow cytometry of CFSE-stained THP-1 cells recovered from the peritoneal cavity of recipient mice 24 h later to assess NK cell killing activity *in vivo*. To facilitate the identification of THP-1 cells *vs* NK92 cells, recovered cells were stained with CD56-APC for the selective labeling of NK92 cells. The representative flow cytometry profile **(B)**, summary graph showing percentage (**(C)**, *left*), and ratio (**(C)**, *right*) of residual THP-1 cells. Data are shown as the mean ± SD. *P < 0.05; Student′s *t*-test.

To verify the effect of forced NKp30 expression *in vivo* in the context of IL-2 deprivation, a xenograft mouse model with THP-1 cells was established (**Figure 7A**). Immune-deficient NSG mice lacking NK cells were i.p.-injected with CFSE-labeled THP-1 cells and subsequently administered with wild-type NK92 cells or NK92 cells overexpressing NKp30a (ΔNCR3+NKp30a) at an E:T ratio of 2:1 without IL-2 supplementation. Analysis of the peritoneal fluid by flow cytometry revealed that the THP-1 cells were eliminated more efficiently by NKp30a-expressing NK92 cells than wild-type NK92 cells (**Figures 7B, C**). The numbers and percentages of residual THP-1 cells were significantly reduced by NKp30a-expressing NK92 cells compared with wild-type NK92 cells. Thus, these results suggested that NKp30 overexpression in NK cells could enhance the NKp30-dependent anti-tumor activity and improve the *in vivo* therapeutic potency of NK cells without IL-2 supplementation.

## Discussion

In this study, we demonstrated that IL-2 deprivation impaired NK cell cytotoxicity by preferentially downregulating NKp30 expression, which could be rescued by the overexpression of an immunostimulatory NKp30 isoform such as NKp30a or NKp30b. The results support the essential role of IL-2 supplementation in the sustained expression of NKp30 on activated NK cells; NKp30 is a critical activating receptor in triggering the NK cell-mediated killing of diverse tumor cells and a prognostic biomarker in tumors including AML. In support, using IL-2-dependent NK92 and IL-2-independent NK92MI cells, we observed that NK cell cytotoxicity against K562 and THP-1 AML cells, besides NKp30-mediated cytotoxicity, was severely impaired by IL-2 deprivation. In particular, the therapeutic relevance of this finding was supported by our identification of an improved clearance of THP-1 cells by NK92 cells overexpressing NKp30a *in vivo* without IL-2 supplementation. Treatment with IL-2 upregulates NKp30 expression on freshly isolated peripheral NK cells (27, 46), whereas the same treatment maintained the upregulated expression of NKp30 on primary expanded NK cells adopted in clinical trials (**Figure 1** and data not shown). Thus, our results suggest that IL-2 supplementation plays an important role in regulating cytotoxicity through the retention of the expression of activating receptors on NK cells, particularly NKp30, besides the previously appreciated NK cell survival and proliferation. In this regard, further study on the dependence of NKp30 among other receptors on IL-2 would be required to validate our results with the inclusion of more donors and patient cases. Considering that IL-2 toxicity has limited widespread application in clinical trials, NKp30 upregulation, as shown here by the ectopic overexpression, may provide a novel modality to harness NK cells in cancer immunotherapy.

NKp30 is constitutively expressed on NK cells to recognize B7-H6, BAT3, and heparin sulfate (45). The signal upon the engagement with the ligands is transduced through the FcϵRIγ and CD3ζ adaptor molecules. Among the six alternatively spliced isoforms of NKp30, the function of NKp30a, NKp30b, and NKp30c are most known; NKp30a and NKp30b are immunostimulatory, while NKp30c is immunosuppressive. Gastrointestinal stromal tumor (GIST) patients expressing NKp30a or NKp30b as the most abundant isoform exhibited significantly better overall survival compared to the patients with higher NKp30c isoform expression (47). Moreover, high *NCR3* and NKp30a absolute expression levels are positive prognostic biomarkers in patients with non-small cell lung cancer (48). NKp30 expression is downregulated on peripheral NK cells in AML patients (49) and on tumor-infiltrating NK cells in GIST patients (47). NKp30 signaling can be inhibited by pp65 of human cytomegalovirus (50) and CEACAM1 (51). Following donor-derived NK cell infusion after haploidentical hematopoietic cell transplantation four times on days 6, 9, 13, and 20, the higher expression level of NKp30 on donor NK cells is an independent predictor of enhanced complete remission and reduced leukemia progression, although early donor NK cell infusion does not impede leukemia progress significantly (52). Furthermore, a high expression level of NKp30 was related to significantly better overall and relapse-free survival compared with the low expression level of NKp30 in patients with intermediate-risk AML (44). Nonetheless, the evidence for the expression of NKp30 under the regulation of IL-2 has been less elucidated so far in the context of human NK cell cytotoxicity against tumor cells.

In addition to IL-2, IL-15 in culture upregulates NKp30 expression on peripheral NK cells from healthy volunteers and AML patients (46). In comparison, the expression of NKp46 on NK cells was not significantly affected by the treatment with IL-2 and IL-15. In this study, we observed that IL-15 deprivation led to a significant downregulation of NKp30 but not NKp46 as did IL-2 deprivation (**Supplementary Figure S1**), compatible with a finding that IL-2 and IL-15 receptors share the β subunit and common γ chain for signaling (38). IL-15 induces NKp30 expression on human circulating CD8^+^ T cells, which exert an NK-like anti-tumor effect (53). The NKp30^+^CD8^+^ cells also express FcϵRIγ, and FcϵRIγ expression requires Syk and PLZF (53). In humans, FcϵRIγ^-/low^ NK cells have reduced expression levels of NKp30, NKp46, PLZF, and Syk. The FcϵRIγ^-/low^ NK cells exhibit downregulated mTOR activity, and FcRγ upregulation is dependent on IL-2 or IL-15 (40). FcϵRIγ deletion using CRISPR-Cas9 abolishes the expression of NKp30 and NKp46, supporting the role of FcϵRIγ in NKp30 expression (54). In brief, IL-2 and IL-15 upregulate FcϵRIγ expression though mTOR activity, and consequently NKp30 expression (40). Compatible with this finding, we observed a moderate decrease in FcϵRIγ expression (~29%) and, in particular, a considerable decrease in NKp30 expression (~77%) by IL-2 deprivation (**Supplementary Figure S2**). Thus, we speculate that ectopic overexpression of NKp30 can improve an impaired cytotoxicity of NK cells by IL-2 deficiency likely due to the relative insensitivity of FcϵRIγ expression to IL-2 deprivation, which merits further investigation. Despite the common requirement of FcϵRIγ for the surface expression of NKp30 and NKp46 (34), the expression level of NKp46 did not decrease but rather increased due to deprivation of IL-2 and IL-15 in our study, unlike that of NKp30. IL-2 deprivation likely occurs during NK cell adoptive therapy without IL-2 support *in vivo* or in the TME, partly due to regulatory T (Treg) cells (55, 56). This discordant regulation between NKp30 and NKp46 by IL-2 deprivation is compatible with a recent study on the sustained expression of NKp46 but the downregulation of other activating receptors, such as NKp30, NKG2D, and NKp44, in tumor-infiltrating NK cells in diverse leukemia and tumors (49, 57, 58). In this regard, we do not exclude alternative mechanisms to regulate the expression of NKp30 compared to that of NKp46, which merits further investigation.

IL-2 is a quintessential cytokine for NK cells to survive and proliferate, hence low dose and ultra-low dose IL-2 therapies have been tried in a clinical setting for patients with various tumors. However, the toxicity and side effects of IL-2 have limited the application of such therapies. Genetically engineered IL-2 molecules are initially developed to reduce the toxicity and expansion of Treg cells. A mutant IL-2 (F42K) that binds to IL-2Rβγ with the lower affinity promoted NK cell activation but still induces the expansion of Treg cells in a mouse melanoma model (59). Fucoidan-based IL-2 delivery microcapsules enhanced adoptive T cell therapy in a mouse melanoma model (60). Nonetheless, the capacity of IL-2 to augment Treg cells is one of the major concerns in immunotherapy. Even ultra-low doses of long-lived IL-2 can induce the prolonged increase of Treg cells in non-human primates (61). Our study suggests NKp30 upregulation as an alternative strategy to improve NK cell immunotherapy, possibly with a combination of newly developed safer IL-2 delivery, as our results showed that the upregulation of NKp30 expression enhanced NK cytotoxicity, particularly NKp30-mediated cytotoxicity, even in the presence of IL-2. In this respect, it requires further investigation on the development of a practical and safe way to upregulate NKp30 such as the genetic engineering to overexpress NKp30 as shown here and the selection of superior donors with the high-level expression of immunostimulatory NKp30 isoform on NK cells.

The current results indicated that NKp30 overexpression in the absence of IL-2 supplementation could improve NK cytotoxicity against THP-1 AML cells expressing moderate levels of the NKp30 ligand B7-H6. In addition, regardless of IL-2 supplementation, NK cells efficiently eliminated the K562 leukemic cells highly expressing B7-H6. B7-H6 downregulation renders U-937 leukemic cells resistant to NK cell-mediated cytotoxicity (62). B7-H6 is frequently expressed on diverse tumor cells and upregulated by the proto-oncogene Myc (63) and endoplasmic reticulum stress (64). The expression of NKp30 on NK cells from AML patients is downregulated in the presence of AML cells and does not recover in the culture with IL-2 (49, 65). Thus, the upregulation of NKp30 expression, as shown here by the ectopic overexpression, may be an alternative and viable strategy to improve the outcomes of NK cell immunotherapy for patients with cancer, particularly AML, without potential risks of IL-2 infusion.

## Data availability statement

The original contributions presented in the study are included in the article/**Supplementary Material**, further inquiries can be directed to the corresponding author.

## Ethics statement

Human primary samples were obtained from healthy donors after informed consent in accordance with protocols approved by the Institutional Review Board of Asan Medical Center and the Declaration of Helsinki (2018-0611). The studies were conducted in accordance with the local legislation and institutional requirements. The participants provided their written informed consent to participate in this study. The animal study was approved by the Institutional Animal Care and Use Committee of the Asan Institute for Life Sciences. The study was conducted in accordance with the local legislation and institutional requirements.

## Author contributions

NK: Writing – original draft, Writing – review & editing. EY: Data curation, Formal analysis, Investigation, Methodology, Writing – review & editing. EL: Data curation, Investigation, Methodology, Writing – review & editing. HP: Data curation, Investigation, Methodology, Writing – review & editing. HK: Writing – original draft, Writing – review & editing.
