# Supplementary material for: Interleukin-2 is required for NKp30-dependent NK cell cytotoxicity by preferentially regulating NKp30 expression
Source: Front Immunol. 2024 Apr 18;15:1388018. doi: 10.3389/fimmu.2024.1388018 (PMC11063289; doi:10.3389/fimmu.2024.1388018)
Supplement: Supplementary file 1 [file DataSheet_1.docx]

**Supplemental figures**

**
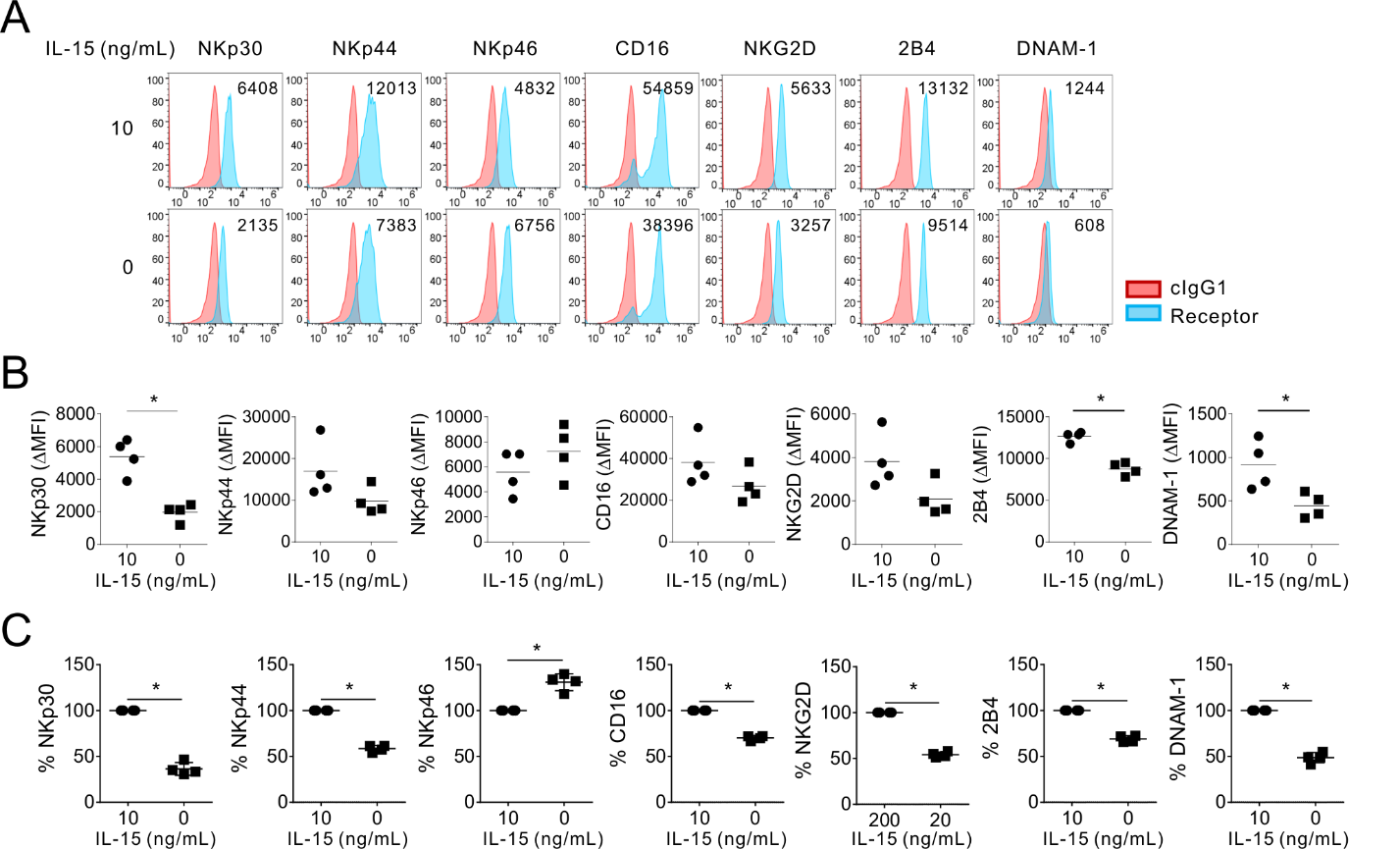
**

**Fig. S1. IL-15 depletion preferentially affects the surface expression of NKp30.**

Primary expanded NK cells were incubated in the absence or presence of IL-15 (10 ng/mL) for 24 h, and the surface expression of NKp30, NKp44, NKp46, CD16, NKG2D, 2B4, and DNAM-1 on NK cells was determined by flow cytometry (*n* = 4 per group). (**A**) Representative FACS profiles showing the MFI of the indicated receptor expression (blue shaded histograms) on NK cells. Isotype control staining is shown as red shaded histograms. (**B**) Summary graphs showing the MFI of the indicated receptors on NK cells relative to the MFI of the isotype control (ΔMFI). Horizontal bars denote the medians. (**C**) Shown is the normalized levels for the expression of the indicated receptors. [% MFI = (ΔMFI of each condition/ΔMFI of IL-15 10 ng/mL) × 100]. Horizontal bars denote the medians. **P* < 0.05; Mann-Whitney *U* test. Each data point represents a single donor.


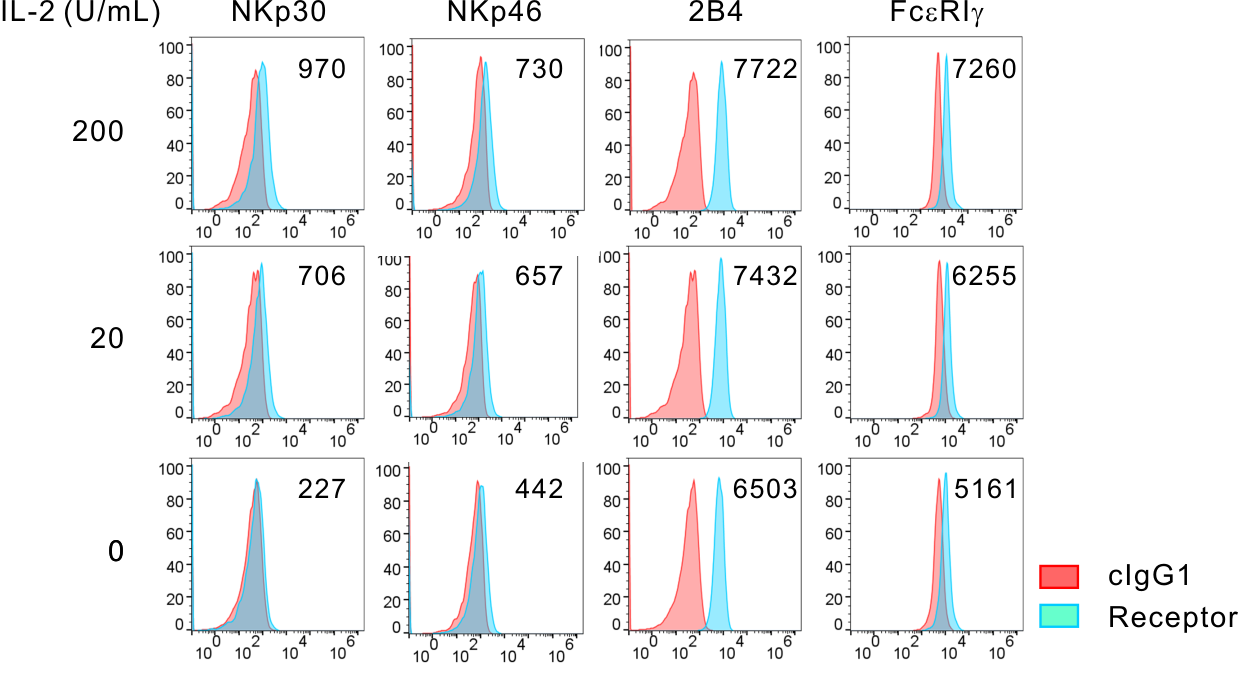


**Fig. S2. IL-2 depletion leads to the downregulation of FcεRIγ in NK92 cells.**

NK92 cells were incubated with the indicated dose of IL-2 (0, 20, or 200 U/mL) for 12 h and were used to determine the surface expression of NKp30, NKp46, 2B4, and FcεRIγ by flow cytometry. Representative FACS profiles showing the MFI of the indicated receptor expression (blue shaded histograms) on NK92 cells relative to the MFI of the isotype control (ΔMFI). Isotype control staining is shown as red shaded histograms.


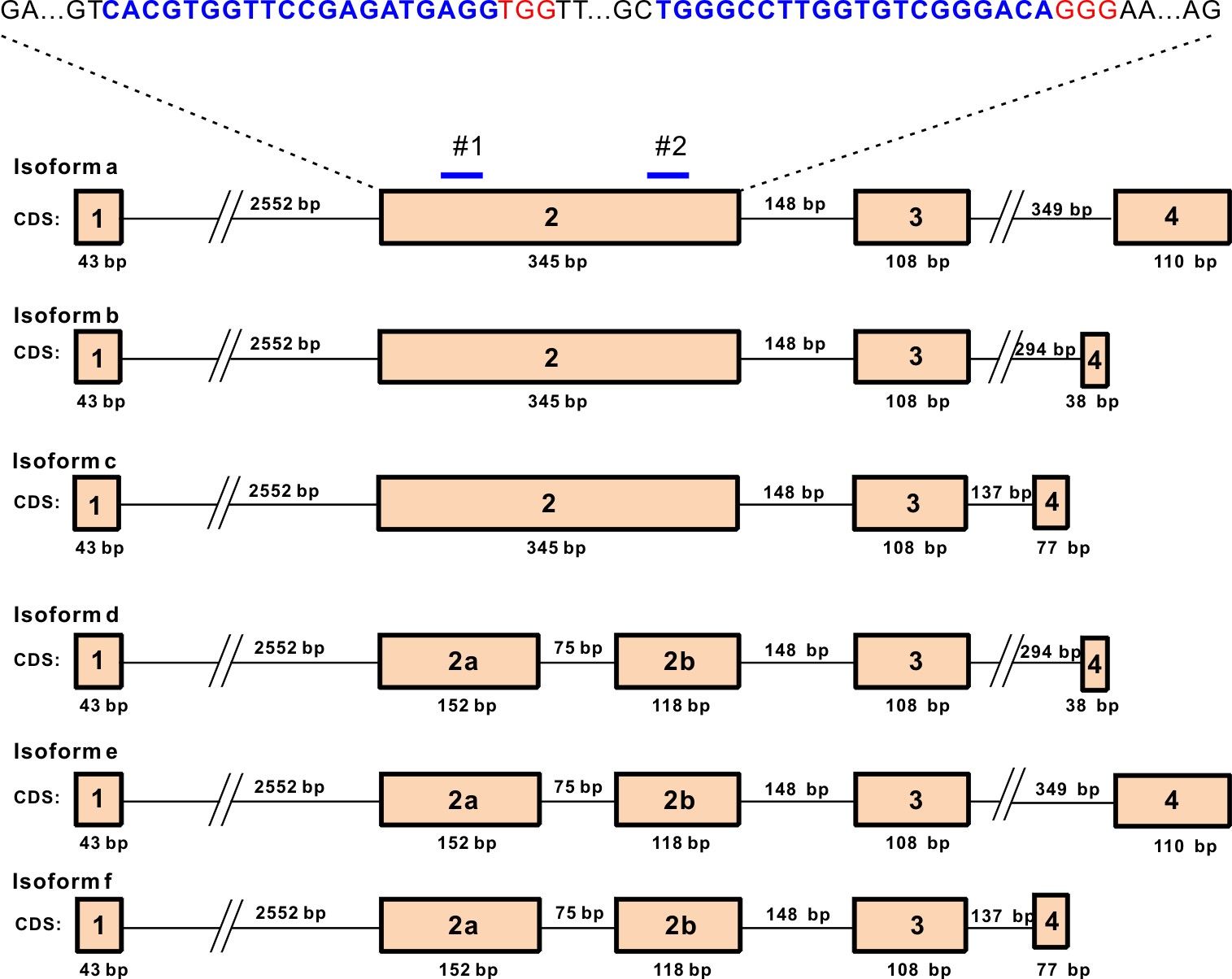


**Fig. S3. CRISPR/Cas9-mediated knockout of NKp30.**

Scheme of CRISPR/Cas9-mediated knockout of NKp30 using specific gRNA #1 or gRNA #2. NKp30 has six variants (NKp30a, NKp30b, NKp30c, NKp30d, NKp30e and NKp30f), attributed to alternative splicing. To delete all six variants of NKp30, two gRNAs with target sequences that are common in exon 2 were selected. The target sites of the selected gRNAs are indicated in the blue bar, along with the 20-nt target sequences (blue). The PAM sequence is denoted in red.


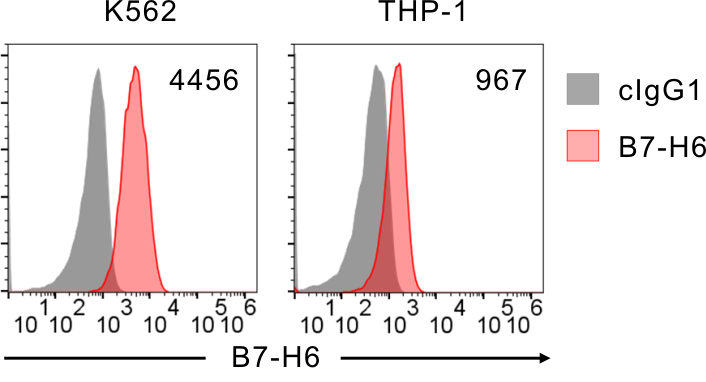


**Fig. S4. Expression of NKp30L (B7-H6) on K562 and THP-1 cells.**

The surface expression of B7-H6, the ligand of the NKp30 receptor, was analyzed by flow cytometry on K562 and THP-1 cells (red shaded histograms). Isotype control staining is shown as gray shaded histograms. Shown number is the MFI of the B7-H6 on the cells relative to the MFI of the isotype control (ΔMFI). Data are representative of at least three independent experiments.


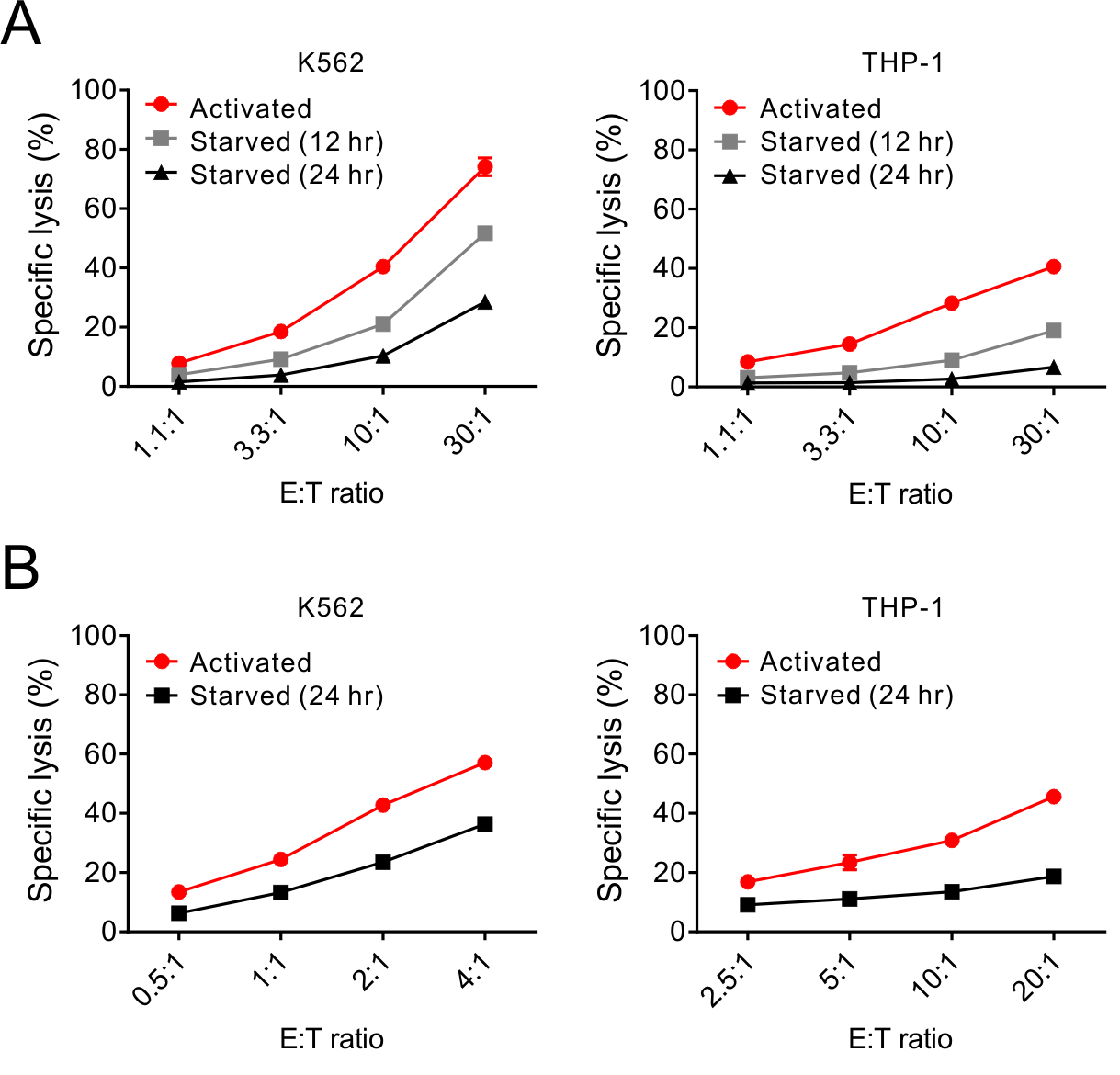


**Fig. S5. IL-2 depletion in NK cells leads to compromised cytotoxicity against K562 and THP-1 cells.**

(**A**-**B**) NK92 cells (**A**) or primary expanded NK cells (**B**) were incubated with (activated) or without (starved) IL-2 (200 U/mL) for the indicated times and were then mixed with K562 or THP-1 cells. After incubation for 2h, NK cell cytotoxicity was assessed using europium-based cytotoxicity assay at the indicated effector to target (E:T) cell ratios. Data are shown as the mean ± SD. Data are representative of at least three independent experiments.


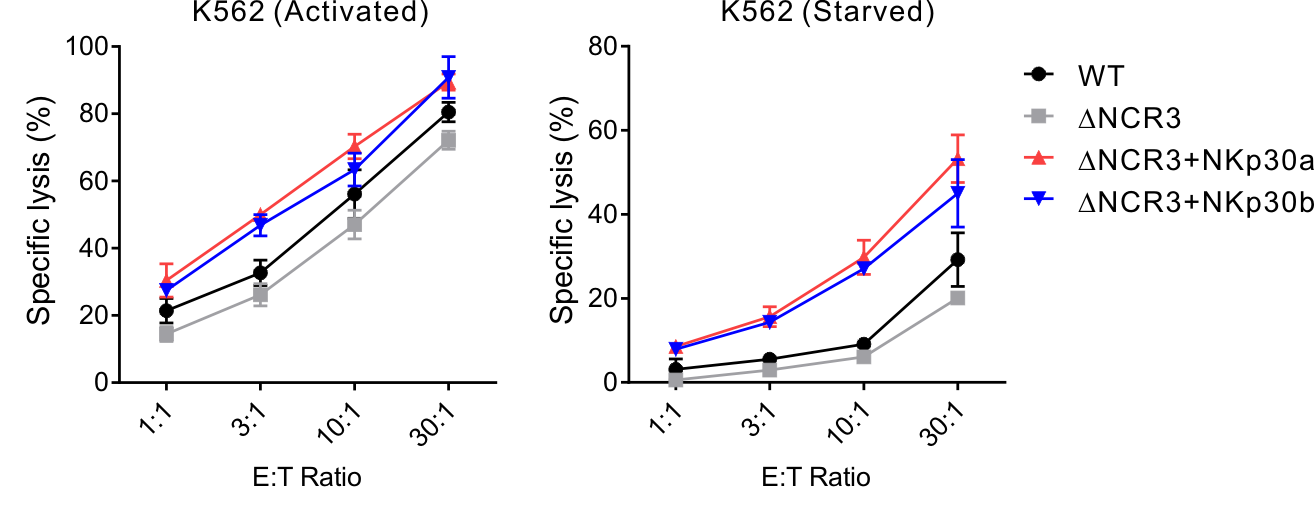


**Fig. S6. NKp30 overexpression restores NK cell responsiveness to K562 cells in IL-2 deficient condition.**

NK92-WT, ΔNCR3, ΔNCR3+NKp30a, and ΔNCR3+NKp30b cells were incubated with (activated) or without (starved) IL-2 (200 U/mL) for 24 h and were then mixed with K562 cells. NK cell cytotoxicity was determined after 2 h using europium-based assay at the indicated effector to target (E:T) cell ratios. Data are shown as the mean ± SD. Data are representative of at least three independent experiments.
